# Hypothyroidism Due to Hepatic Hemangioendothelioma: A Case Report

**DOI:** 10.4274/jcrpe.v2i3.126

**Published:** 2010-08-07

**Authors:** Semra Çetinkaya, Havva Nur Peltek Kendirici, Sebahat Yılmaz Ağladıoğlu, Veysel Nijat Baş, Sonay Özdemir, Ceyhun Bozkurt, Zehra Aycan

**Affiliations:** 1 Pediatric Endocrinologist, Dr. Sami Ulus Obstetrics and Gynecology, Children's Health and Disease Training and Research Hospital, Department of Pediatric Endocrinology, Ankara, Turkey; 2 Pediatric Oncologist, Dr. Sami Ulus Obstetrics and Gynecology, Children's Health and Disease Training and Research Hospital, Department of Pediatric Oncology, Ankara, Turkey; +90 505 388 44 03+90 312 317 03 53semcetinkaya@gmail.comPediatric Endocrinologist, Dr. Sami Ulus Obstetrics and Gynecology, Children's Health and Disease Training and Research Hospital, Department of Pediatric Endocrinology, Ankara, Turkey

**Keywords:** Hepatic hemangioendothelioma, consumptive hypothyroidism, type 3 iodothyronine deiodinase

## Abstract

Although hemangioendothelioma (HHE) is a commonly encountered hepatic tumor during infancy, HHE−related hypothyroidism is rare. We present a patient who developed HHE−related hypothyroidism during the neonatal period and showed marked improvement in hypothyroidism by regression of HHE. A 28−day−old boy with TSH level of 77 mIU/mL on neonatal screening and diagnosed as congenital hypothyroidism was started on L−thyroxine (L−T4) (11 μg/kg/day) therapy on the 21^th^ day of life. On physical examination, the liver was palpable 5 cm below the right costal margin, and the thyroid gland was nonpalpable. Thyroid ultrasonography was normal. Although L−T4 dose was increased to 15 μg/kg/day, TSH was not suppressed and free T3 level remained low. HHE in both lobes of the liver was detected by abdominal ultrasonography and magnetic resonance imaging. Treatment was started with prednisolone 2 mg/kg/day and alpha−interferon 3 million U/m^2^/3 times per week. Thyroid dysfunction was thought to be due to type 3 iodothyronine deiodinase activity expressed by HHE. L−T4 therapy was changed to Bitiron^®^ tablet, which includes both T4 and T3, and euthyroidism was attained within 1 month. Thyroid hormone requirement was reduced and treatment was discontinued after regression of the HHE. At the most recent visit, the patient was 21 months old and off treatment. His growth and neurological development were normal for age and he was euthyroid. HHE should be considered in cases with severe hypothyroidism resistant to high−dose thyroid hormone replacement. The treatment of HHE in combination with T4 and T3 therapy results in euthyroidism.

**Conflict of interest:**None declared.

## INTRODUCTION

Hepatic hemangioendothelioma (HHE) is a common tumor of the liver in infancy (1). Although benign, these tumors may cause complications such as cardiac failure, coagulation disorders and, rarely, hypothyroidism (1, 2). The association between HHE and hypothyroidism has first been reported in 2000 (3) and there have been very few case reports since then. In HHE, the main pathophysiology can be summarized as an increased type 3 iodothyronine deiodinase activity and catalysis of the conversion of thyroxine (T4) to reverse triiodothyronine (T3), and of T3 to diidothyronine (T2) by this enzyme. Hypothyroidism develops when the rate of inactivation of thyroid hormones surpasses the rate of their production, and this phenomenon is known as "consumption hypothyroidism" (1, 3, 4, 5). This hypothyroidism is resistant to thyroid replacement therapy and is characterized by low free T3 (fT3) and high reverse T3 (rT3) levels despite the high TSH and relatively high free T4 (fT4) levels (5). High doses of Na L−thyroxine (L−T4) are required to normalize these parameters (3). It is possible to keep the Na L−T4 dose lower by administering T4 and T3 together during treatment. However, the ideal option is the resolution of HHE by treatment or spontaneously (5).

We present a case initially diagnosed as hypothyroidism in the newborn period, but later as HHE when it was found that Na L−T4 treatment in standard doses remained inadequate to correct the hypothyroid state.

## CASE REPORT

A 28−day−old male patient was referred to our clinic with a diagnosis of congenital hypothyroidism. The patient was the product of the 3^rd^ pregnancy of a 36−year−old healthy mother. Birth weight was 3600 g. Based on a TSH value of 77 mIU/mL (N= 0.5−6.5) reported by the newborn screening program when the baby was 21 days old, a diagnosis of congenital hypothyroidism was made and Na L−T4 was started in a dose of 11 μg/kg/day. The patient was referred to our clinic for follow−up.

**Figure 1:**
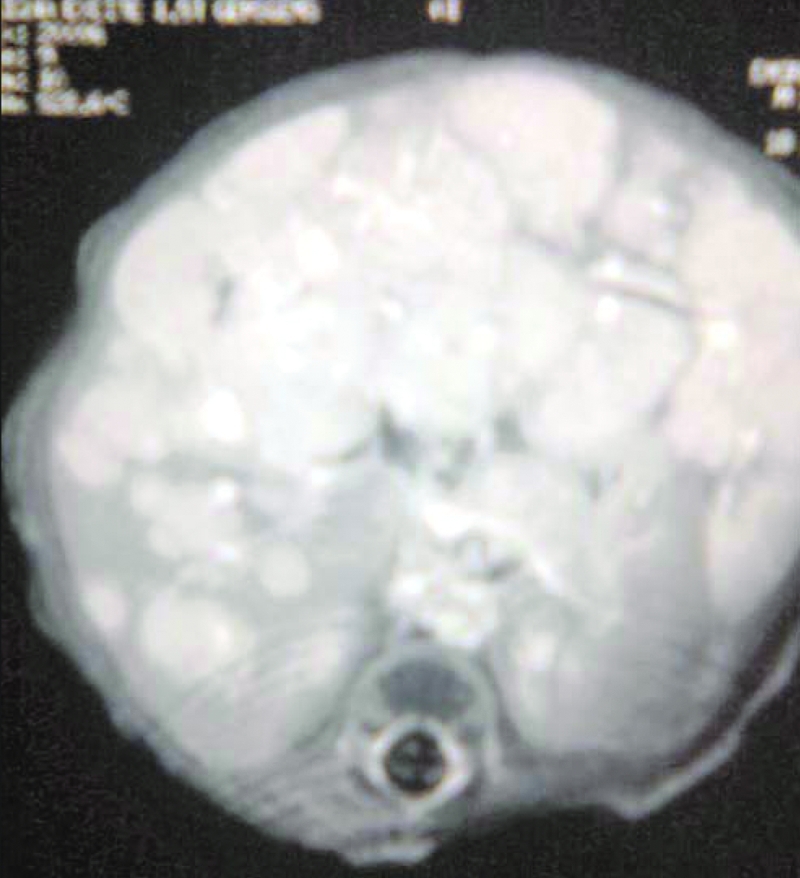
Abdominal MRI showing multiple hepatic nodules

There was no exposure to iodine or any noteworthy health problem in the family history. The patient’s weight was 4500 g, length 52 cm, and head circumference 37 cm. Physical examination revealed mild abdominal distention, prominent superficial veins on the abdominal skin, and an enlarged liver palpable 5 cm below the right costal margin. The thyroid gland was not palpable. The infant had received a standard daily dose of Na L−T4 for a week. Blood hormone levels were: TSH >150 mIU/mL, T4 10.8 μg/dL (5.9−16.3), fT4 2.41 ng/dL (0.9−2.1), T3 0.43 ng/mL (0.94−2.69), and fT3 1.55 pg/mL (2−6.5). Complete blood count and liver function test results were normal. The mother's thyroid function tests were normal and no thyroid autoantibodies were found in the mother or in the baby. Thyroid ultrasonography was normal. The Na L−T4 dose was increased to 15 μg/kg daily. Abdominal ultrasonography showed multiple hypoechoic lesions with vascularization in both lobes of the liver, the largest being 2 cm in size. Abdominal magnetic resonance imaging (MRI) showed multiple hepatic nodules with decreased signal intensity on T1−weighted images and increased signal intensity on T2−weighted images, i.e. findings consistent with a diagnosis of HHE; the largest nodule was 3 cm in size (Figure 1). The alpha−fetoprotein level was high (2667 ng/mL), while the urine vanillylmandelic acid level was within normal limits. Congestive heart failure developed during clinical follow−up and treatment with digoxin and furosemide was started. A contrast echocardiographic investigation showed a pulmonary arteriovenous fistula. The hemangioendothelioma was treated with methylprednisolone in doses of 2 mg/kg/day and alpha−interferon in doses of 3 million U/m^2^/3 times per week. The patient's thyroid function disorder was thought to be due to the activity of the type 3 iodothyronine deiodinase expressed by the HHE, but rT3 level could not be measured due to technical inadequacies. The thyroid treatment was changed to Bitiron^®^ tablets, which contain T4 and T3 together (50 μg of Na L−T4 and 12.5 μg of triiodothyronine sodium). The cardiac failure resolved on the 12^th^ day of treatment. At the end of one month, blood hormone levels were: TSH 4.28 mIU/mL, T4 17.4 μg/dL, fT4 1.85 ng/dL, T3 2.14 ng/mL, and fT3 3.96 pg/mL. Repeat ultrasonographic examinations showed that the hemangioendotheliomas shrank in size, while repeat contrast echocardiographic investigations revealed that the pulmonary arteriovenous fistula had disappeared. The patient was given methylprednisolone for 3 months. Abdominal ultrasonography on the 11^th^ month of alphainterferon treatment showed multiple nodular lesions with hypoechoic−heterogenous structures and ill−defined borders in the liver. The serum alpha−fetoprotein level had decreased to 5.05 ng/mL. At this time, blood hormone levels were: TSH 2.5 mIU/mL, T4 9.54 μg/dL, fT4 1.23 ng/dL, T3 1.75 ng/mL, and fT3 6.46 pg/ml. Thyroid hormone replacement therapy (Na L−T4 0.6 μg/kg/day + Liothyronine 0.15 μg/kg/day) was discontinued. Blood hormone levels were completely normal 2 months later (TSH 1.86 mIU/mL, T4 11.2 μg/dL, fT4 1.22 ng/dL, T3 3.23 ng/mL, fT3 8.11 pg/mL). No recurrence was found at the follow−up 6 months after treatment discontinuation. The patient was last seen at age 21 months. Growth and neurological development were normal and he showed no signs of thyroid deficiency. Table 1 summarizes the clinical and laboratory findings of the patient.

**Table 1:**
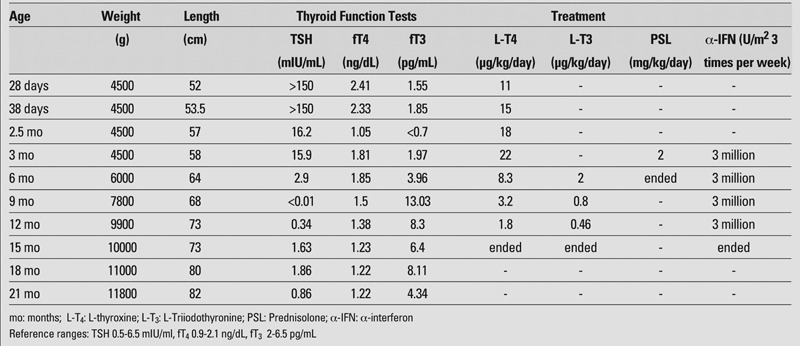
The follow−up clinical and laboratory findings and treatment schedule of the patient

## DISCUSSION

Huang et al (3) were the first to describe an association between severe hypothyroidism requiring high−dose thyroxine support and HHE in an infant. These authors reported that in the HHE tissue, type 3 iodothyronine deiodinase enzyme activity was 7.5 times that of the placenta, while Bessho et al (5) found this level to be more than 10 times that of the placental tissue. The etiology of the increased type 3 iodothyronine deiodinase activity in HHE is not fully known (5). Some immunohistochemical markers, such as the specific HHE marker antiglucose transporter−1 (GLUT1), have been reported to be present in infantile hemangioma and in placental endothelial cells. It has been postulated that HHE may originate from placental angioblasts (6, 7). Mo et al (8) stated that hemangiomas may arise from embolization of placental endothelial cells and that the liver carries great risk regarding hemangioma development as it is the first organ to be perfused with the incoming blood from the placenta. These postulated mechanisms may explain the high activity of type 3 iodothyronine deiodinase in HHE and the self−limiting growth of the tumor (5).

Ayling et al (9) have speculated that HHE may secrete TSH−like factors that can interact with TSH receptors and may be responsible for the thyroid dysfunction. One patient's postmortem liver tissue was stained immunohistochemically with TSH and showed positivity for the TSH beta subunit together with empty follicles that supported increased TSH secretion in the thyroid tissue. Ho et al (10) have similarly reported resistant elevated TSH levels despite normal fT3 and fT4 levels in hepatic hemangioma patients and stated that the tumor tissue may produce a TSH−like factor. The absence of clinical signs of hyperthyroidism in the patient and normal or slightly increased T3 and T4 levels indicate that the TSH−like factor was probably inactive. Increased type 3 iodothyronine deiodinase activity and ectopic TSH−like factor production may together or separately cause thyroid dysfunction in hemangioendothelioma patients (1). The marked increases in serum rT3 concentrations during treatment with L−T4 could be explained by the increase in the activity of type 3 iodothyronine deiodinase (11). Although we did not know the type 3 iodothyronine deiodinase enzyme activity in our patient's hemangioendothelioma tissue and we were unable to measure the rT3 level due to technical inadequacies, we believe that the cause of the severe hypothyroidism in our patients was the increased type 3 iodothyronine deiodinase enzyme activity. This view was supported by the fact that change was observed in the thyroid function tests, which returned to normal when T3 was added to the T4 treatment the patient was taking. Our patient did not have symptoms of hyperthyroidism, such as tachycardia and restlessness, despite the increased Na L−T4 dose, and the thyroid function tests started to return to normal when a T3 preparation was added to the treatment. However, the patient had also started to receive HHE treatment at this stage and the lesions had started to shrink. Although the addition of a T3 preparation may have contributed to the recovery of the thyroid functions, we believe that the real recovery was due to the regressing HHE.

Thyroid dysfunction in HHE is generally related to the severity and distribution of the hepatic hemangiomas (1). Most cases of hypothyroidism are reported to have widespread liver involvement. This may be due to the increased enzyme activity in the large tumor area or to the decreased hepatic inactivation of deiodinase activity with the invasion of the liver parenchyma by the hemangioendothelioma (12). Consistent with this view, we believe that our case developed severe hypothyroidism due to the widespread HHE.

Hypothyroidism due to HHE may not be detected on newborn screening as the lesions usually appear around the 4th to 6th weeks of life. However, HHE cases, which were symptomatic in the neonatal period, have been reported (13). Severe symptoms of hypothyroidism may be masked by HHE complications (3, 12). In our patient, the hypothyroid state was detected with the newborn screening program and HHE was found on presentation to the endocrinology clinic for the follow−up of the hypothyroidism. It is well known that thyroid hormones are necessary for growth and development during early infancy and that the early detection and treatment of hypothyroidism is important to prevent growth retardation and intellectual loss (5, 10, 12). The normal motor and mental development noted in our patient may be explained with the early diagnosis and treatment of the hypothyroidism.

A thyroxine dose of 5−10 μg/kg/day is generally adequate for treating congenital hypothyroidism, while very high doses of T4 and T3, such as 22−70 μg/kg/day, are necessary to normalize the serum TSH concentration in children with consumption hypothyroidism and these doses may have to be administered intravenously at times (4,5). Huang et al (3) have reported that a thyroid hormone dose up to 8−9 times that accepted for replacement in congenital hypothyroidism due to thyroid agenesis is required in such cases. The high replacement dose required and the increased rT3 level are thought to reflect tumor size and type 3 deiodinase activity (4). In addition to the hormone replacement treatment, the spontaneous or treatment−related regression of HHE is also important in the normalization of thyroid hormone levels (5). In our patient, the L−T4 dose had to be increased to as high as 22 μg/kg/day. From the 15th month on, with regression of the HHE following the steroid and alpha−interferon treatment, the patient had no need for any treatment.

Although spontaneous recovery may be seen in some HHE cases, the development of serious complications may require treatment such as steroids, alpha−interferon, hepatic artery ligation, radiotherapy, chemotherapy, surgical resection, and liver transplantation (14). There is no consensus on the treatment and management of hemangioendotheliomas, but there are practical clinical algorithms for the evaluation and management of this condition (15, 16). Post−treatment recurrences have been reported although HHE is thought to show self−limiting growth (5). Long−term follow−up of the patients is therefore important. The HHE in our case resolved with the 3−month steroid and 1−year alpha−interferon treatment and there was no recurrence in the first 6 months following treatment discontinuation (Table 1).

Various growth factors that play a role in the proliferative phase of the hemangioma have been defined. Animal studies have shown the type 3 iodothyronine deiodinase enzyme activity that is stimulated with these growth factors to be inhibited by dexamethasone and that the consumption effect could therefore be reduced (17). However, some researchers state that since steroids increase the conversion of T4 to T3 by disturbing type 1 deiodinase activity and inducing type 3 deiodinase activity, they may therefore increase the thyroid hormone requirement (4). It is difficult to evaluate the effect of the steroid treatment on thyroid hormone requirement in our patient, since high−dose thyroid hormone was used concomitantly with the steroid treatment.

In conclusion, we believe that HHE should be considered and investigated when TSH is high, fT3 low and rT3 high despite high−dose L−T4 treatment in congenital hypothyroidism. Our experience with this patient has shown that thyroid functions can recover with HHE treatment and that it is more appropriate to use L−thyroxine and triiodothyronine (T4+T3 preparation) concomitantly to achieve euthyroidism during this period.

**Table 1:**
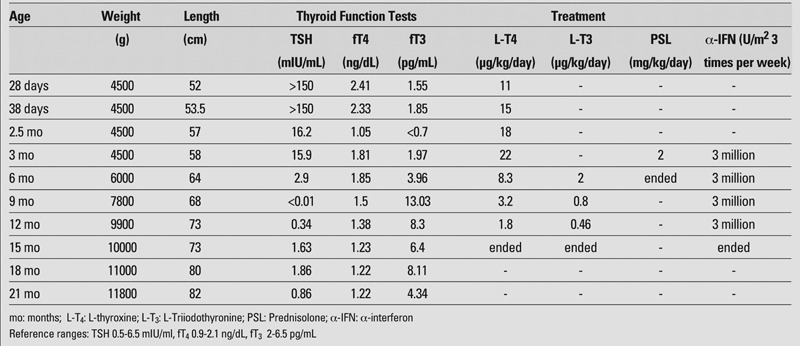
The follow−up clinical and laboratory findings and treatment schedule of the patient
